# Quantitative Assessment of Posterior Maxillary Arch for Orthodontic Miniscrew Insertion Using Cone Beam Computed Tomography: A Cross-Sectional Analysis

**DOI:** 10.1155/2022/8257256

**Published:** 2022-05-26

**Authors:** Solmaz Valizadeh, A. Hamid Zafarmand, Sara Hassan Yazdi, Mitra Ghazizadeh Ahsaie

**Affiliations:** ^1^Department of Oral and Maxillofacial Radiology, School of Dentistry, Shahid Beheshti University of Medical Sciences, Tehran, Iran; ^2^Department of Orthodontics, School of Dentistry, Shahid Beheshti University of Medical Sciences, Tehran, Iran; ^3^DDS, School of Dentistry, Shahid Beheshti University of Medical Sciences, Tehran, Iran

## Abstract

**Methods and Materials:**

Cone beam computed tomography records of 35 patients (70 quadrants) from maxilla were evaluated. The images were analyzed using the NNT viewer software (version 23). The measurements were made on axial sections at 2, 4, 6, and 8 mm from CEJ. The optimal sites were defined in terms of mesiodistal palatal or buccal interradicular distance, alveolar cortical bone thickness, and palatal or buccal safe depth of the bone for miniscrew insertion. Descriptive statistics, paired *t*-test, and repeated measure ANOVA were used to analyze the data.

**Results:**

The mean buccal interradicular distance was the lowest between first and second molar (2.44 mm) and the highest between first and second premolar (3.28 mm). The mean palatal interradicular distance was the lowest between first and second premolar (3.64 mm) and the highest between second premolar and first molar (5.30 mm). The mean buccal safe depth was the lowest between canine and first premolar (1.96 mm) and the highest between first and second molar (2.61 mm). The mean palatal safe depth was the lowest between second premolar and first molar (3.35 mm) and the highest between first and second molar (3.56 mm). The thinnest and thickest buccal cortical thicknesses were detected on canine and first molar (1.04 mm) and on the second premolar and second molar (1.56 mm).

**Conclusion:**

The quantity and quality of the maxillary alveolar process is an important factor to decide where to insert the orthodontic miniscrews, necessitating careful preoperative evaluation.

## 1. Introduction

Orthodontic miniscrews also known as microscrews, mini-implants, or TADs (temporary anchoring devices) are intraoral devices specifically designed to be mounted within the bones of jaw in order to provide absolute anchorage [1]. Not needing patient's compliance, today, this technique is widely being used due to its high clinical efficacy, ease of insertion, low cost, and reduction of unwanted movement of other teeth [2, 3]. Applications of these devices are mesialization, distalization, extrusion or intrusion of teeth, space opening, and, in some cases, alignment of midline or inclined plane [4, 5]. Miniscrews are biocompatible, mostly made of titanium or stainless steel. Resistant to load and corrosion, these devices have various length and diameter which could be chosen according to the available space at the site of insertion [6, 7]. The decision on where to place the miniscrew depends on the patients' situation and orthodontic treatment plan. Miniscrews can be inserted in the alveolar part of the maxilla and the mandible between the roots of the tooth of posterior to them, in palate, chin, or under the nasal floor [8, 9].

Various studies have mentioned complications in applications of miniscrews [6, 10, 11]. Lack of initial stability in the bone is one of the main reasons for miniscrew failures. This could be due to insufficient attention to the quality and quantity of the bone at the site of insertion and weak bone-screw interface. Baumgaertel indicated that cortical bone thickness is in charge of primary anchorage capacity of miniscrews, whereas the cancellous bone has a little anchorage effect [12]. In addition, improper angle or location of miniscrew placement may result in contact to tooth root, root perforation, and sinus floor perforation. Inadequate support from the bone around the miniscrews can result in insufficient osseointegration and lack of stability.

In order to insert implant fixtures into the posterior region of maxilla, a thorough clinical examination and radiographic assessments are needed. These examinations help to assess the quality and quantity of the bone and the available space between roots. Conventional radiography such as periapical radiography and panoramic view can present a general information regarding the selected anchorage site [13]. In the study of Tepedino et al. [7], the average interradicular distance was measured using periapical radiographs. However, due to the two-dimensional nature of conventional radiographs, these assessments could be mistaken especially in cases of crowding, dilacerations, and superimpositions. Cone beam computed tomography (CBCT) is a three-dimensional radiography which can accurately define the shape, morphology, and quantity of the maxillofacial bone [14–16]. This imaging modality presents cross-sectional views which can exactly indicate ridge height and width, concavity, angulation degree, the location and shape of tooth root, distance to anatomic landmarks, and bone density [3, 6]. Using CBCT, Haddad et al. indicated higher prevalence of miniscrew failure within the posterior maxilla due to reduced cortical bone thickness and suggested more apical insertion of miniscrew in order to access a denser buccolingual and palatal bone level [17]. However, in another study, it was shown that the palatal aspect of maxilla contained enough cortical bone thickness and depth to provide sufficient anchorage for miniscrews [12].

This study aimed at determining the optimal sites of miniscrew insertion in the buccal and palatal alveolar cortical bone in posterior maxilla by determining cortical bone thickness, interradicular distance, and safe depth of miniscrew insertion using CBCT imaging.

## 2. Methods and Materials

This was a retrospective cross-sectional study. The study protocol was approved by the Institutional Review Board of Shahid Beheshti University of Medical Sciences (code no: IR.SBMU.DRC.REC.1398.130), and it was conducted in accordance with the Declaration of Helsinki and its subsequent revisions. The study was conducted in accordance with the STROBE statement.

### 2.1. Sample Size

According to the results of a previous studies [11, 17], the sample size was calculated to be 70 posterior sextant, using the following formula (*z* = 1.96, *d* = 0.25, SD = 1.09):

(1) $$\begin{array}{c} n=\frac{\left(z^{2}{\text{SD}}^{2}\right)}{d^{2}},\;\\ n=\frac{{\left(1.96\right)}^{2}{\left(1.09\right)}^{2}}{{\left(25\%\right)}^{2}}\approx 70. \end{array}$$

Due to the retrospective nature of the study, the selection bias was an issue. To minimize the effect of selection bias, 700 CBCT scans from available data base were assessed for eligibility; out of which, 35 CBCT scans containing a total of 70 posterior maxillary sextants were selected randomly according to the eligibility criteria. Included cases were assessed by an expert oral and maxillofacial radiologist. Demographic information including age and gender were also recorded for each case.

### 2.2. Evaluation of CT Scans

This study was conducted on CBCT scans retrieved from the archives of one central oral and maxillofacial radiology clinic in Tehran, Iran. The CBCT scans acquisition was done by the New Tom VGI CBCT scanner (Quantitative radiology, Verona, Italy) with the exposure settings of 110 kVp and 3.3–20 mA. The size of field of view was determined according to the patients' size and referral reason. Images were evaluated with the maxillary occlusal plane parallel to the horizontal axis using NNT viewer application (version 23).

### 2.3. Inclusion Criteria

1. Dentate patients between 19 and 45 years old, with CBCT scan orders of complete maxillary arch from 2018 to 2020
2. Patients must be dentate in canine, premolars, and molars areas
3. Images must have been of adequate resolution/diagnostic quality

### 2.4. Exclusion Criteria

**Figure 1:**
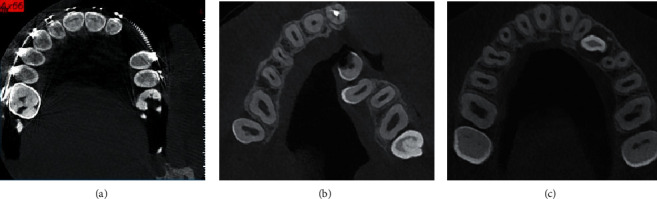
Example of excluded samples. (a) Orthodontic treatment, (b) cleft palate, and (c) impacted canine.

1. Any scan that did not satisfy any of the requirements listed in the inclusion criteria
2. Edentulous patients
3. Presence of severe root dilacerations and anomalies
4. Presence of periodontal disease and bone defects
5. Any scan that included maxillofacial trauma, orthognathic surgery, history of orthodontic treatment congenital anomalies or pathologies such as cysts or tumors, impacted teeth, intraoral exostoses, dental implant, or bone graft (Figure 1)
6. Patients receiving medications affecting the bone metabolism such as bisphosphonate drug, osteoporosis, or other diseases affecting the bone quality and quantity

### 2.5. Measurements

CBCT scans were evaluated by a calibrated oral and maxillofacial radiologist with 20 years of clinical experience. The primary outcome of this study was to assess the bone quantity of different parts of the posterior maxilla for cortical bone thickness and interradicular distance. For this reason, the following parameters were separately measured for ten teeth (canine to second molar at both sides), using the NNT viewer software with a ruler with 0.1 mm accuracy. There were no restrictions with respect to the use of image enhancement filters. The variables were all measured in millimeters. From each CBCT scan, axial CBCT sections at the level of CEJ were detected. The following measurements were performed at 2, 4, 6, and 8 mm vertical distance from CEJ.

**Figure 2:**
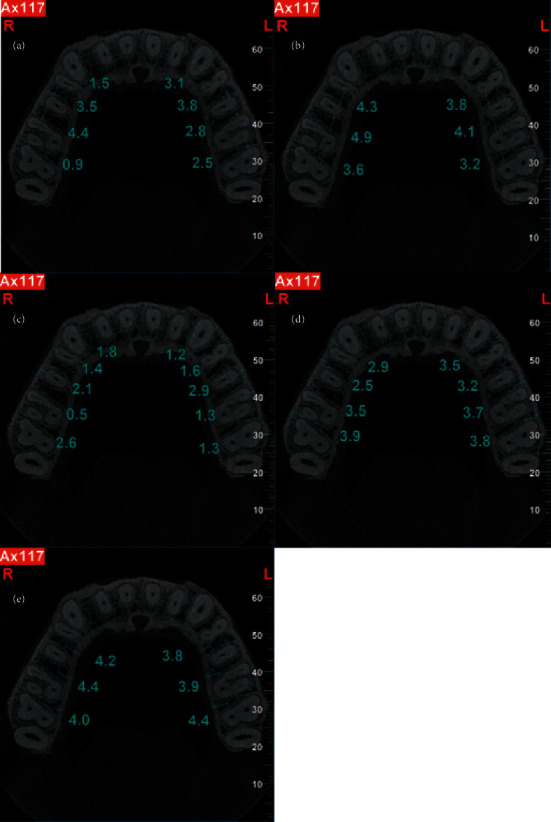
Axial CBCT scans demonstrating measurement (in millimeter) of interradicular distance, cortex thickness, and safe depth of miniscrew insertion. (a) Distance between buccal roots. (b) Distance between palatal roots. (c) Buccal cortex thickness. (d) Safe buccal depth. (e) Safe palatal depth.

1. Distance between buccal roots: the shortest distance between buccal roots of premolars and molars (Figure 2(a)). As canine tooth has one root, the distance between root canine and buccal root of first premolar was measured in this category.
2. Distance between palatal roots: the shortest distance between palatal roots of premolars and molars (Figure 2(b))
3. Buccal cortex thickness: distance between outer buccal cortex to buccal roots of canine, premolars, and molars (Figure 2(c))
4. Safe buccal depth: the distance between the outer cortex of the buccal bone to the narrowest point between buccal roots of premolars and molars (Figure 2(d))
5. Safe palatal depth: the distance between the outer cortex of the palatal bone to the narrowest point between palatal roots of premolars and molars (Figure 2(e))

Subjectivity of measurement was another source of bias in this study. To verify the reliability of measurements, the intraobserver agreement was calculated, using the intraclass coefficient (ICC) test. For this purpose, 20 CBCT scans were evaluated twice with a 1-month interval.

### 2.6. Statistical Analysis

All data were entered into a database system and evaluated using SPSS® for Windows version 21 (SPSS Inc., Chicago, IL, USA, 2012). Patients' data were analyzed anonymously. Every case was assigned a registration number before evaluation to allow explicit and anonymous attribution of necessary information. Data analysis was performed with descriptive statistics, paired *t*-test, and repeated measure ANOVA. The level of significance was set at *p*=0.05.

## 3. Results

### 3.1. Intraoperator Reliability

Measures for the first and second replicates of 20 patients were recorded, and intraclass correlation coefficients (ICC) were established for all measurements. Most measures demonstrated a high degree of reliability between the first and second replicates with ICC values exceeding from 0.81 to 0.98.

### 3.2. Quantitative Assessments

A total of 350 sites were assessed in both left and right sides of the posterior maxilla.

#### 3.2.1. Distance between Buccal and Palatal Roots

**Figure 3:**
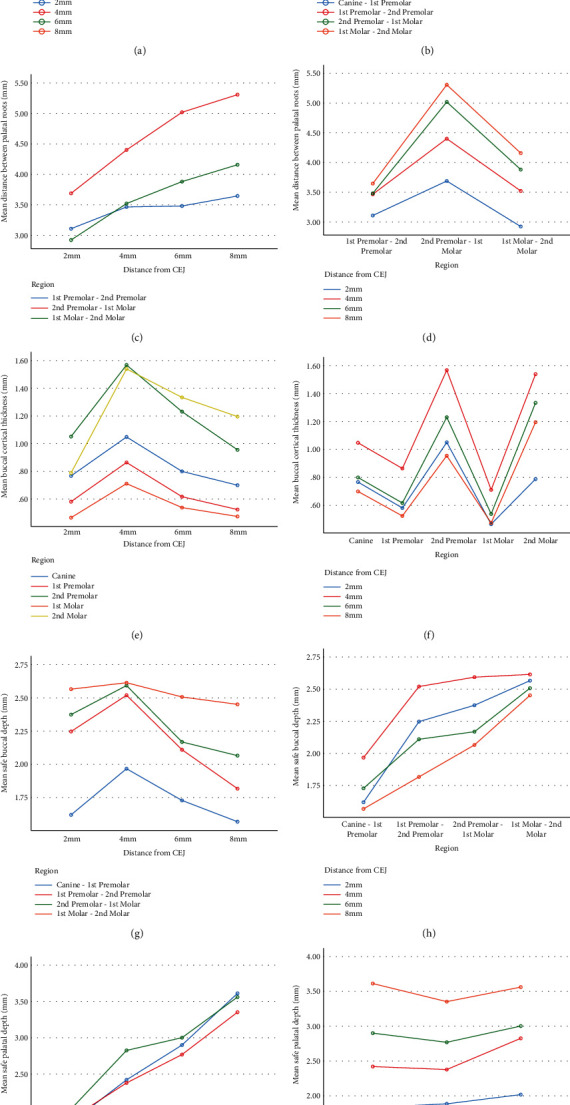
Graphs indicating mean distance between buccal and palatal roots based on region (a and d) and distance from CEJ (b and c). Mean buccal bone thickness based on distance from CEJ (e) and region (f). Mean buccal and palatal safe depth based on distance from CEJ (g and i) and region (h and j).

The mean buccal interradicular distance generally increased from 2 mm from CEJ to 6 mm from CEJ and further decreased from 6 mm from CEJ to 8 mm from CEJ (Figures 3(a)–3(d)). The mean buccal interradicular distance between the canine and first premolar was 2.52 ± 0.86, 2.72 ± 0.81, 2.87 ± 0.90, and 2.73 ± 1.38 at 2, 4, 6, and 8 mm from CEJ. The mean buccal interradicular distance between first and second premolar was 2.75 ± 0.83, 3.01 ± 0.80, 3.28 ± 0.94, and 2.77 ± 1.50 at 2, 4, 6, and 8 mm from CEJ. The mean buccal interradicular distance between second premolar and first molar was 2.71 ± 0.80, 2.79 ± 0.84, 2.87 ± 0.94, and 2.78 ± 1.57 at 2, 4, 6, and 8 mm from CEJ. The mean buccal interradicular distance between first molar and second molar was 2.36 ± 0.91, 2.44 ± 0.85, 2.00 ± 1.09, and 1.64 ± 1.11 at 2, 4, 6, and 8 mm from CEJ (Table 1).

The mean palatal interradicular distance generally increased from 2 mm from CEJ to 8 mm from CEJ and were higher than buccal interradicular distances. The mean buccal interradicular distance between first and second premolars was 3.10 ± 0.59, 3.46 ± 0.69, 3.48 ± 0.89, and 3.64 ± 0.81 at 2, 4, 6, and 8 mm from CEJ. The mean buccal interradicular distance between second premolar and first molar was 3.68 ± 0.90, 4.40 ± 1.09, 5.01 ± 1.08, and 5.30 ± 1.08 at 2, 4, 6, and 8 mm from CEJ. The mean buccal interradicular distance between first molar and second molar was 2.92 ± 0.99, 3.52 ± 0.94, 3.88 ± 1.17, and 4.15 ± 0.95 at 2, 4, 6, and 8 mm from CEJ (Table 1).

#### 3.2.2. Buccal Cortical Thickness

The mean buccal cortex thickness increased from 2 mm to 6 mm CEJ and further decreased from 6 mm to 8 mm CEJ on all posterior teeth in the maxilla (Figures 3(e) and 3(f)). The thickness of the buccal cortex was the highest on second premolar tooth and was the lowest on first molar (Table 2).

#### 3.2.3. Safe Buccal and Palatal Depth

The safe buccal depth increased from 2 mm to 8 mm from CEJ from canine to second molar (Figures 3(g)–3(j)). The mean safe buccal depth between the canine and first premolar was 1.62 ± 0.69, 1.96 ± 0.70, 1.72 ± 0.65, and 1.56 ± 0.74 at 2, 4, 6, and 8 mm from CEJ. The safe buccal depth between first premolar and second premolar was 2.24 ± 1.10, 2.52 ± 0.84, 2.11 ± 0.76, and 1.81 ± 0.76 at 2, 4, 6, and 8 mm from CEJ. The mean safe buccal depth between second premolar and first molar was 2.37 ± 0.93, 2.59 ± 0.81, 2.16 ± 0.80, and 2.06 ± 0.79 at 2, 4, 6, and 8 mm from CEJ. The mean safe buccal depth between first molar and second molar was 1.63 ± 0.99, 2.61 ± 1.03, 2.69 ± 0.86, and 2.45 ± 0.82 at 2, 4, 6, and 8 mm from CEJ The highest buccal depth was detected between first and second molar at 4 mm depth from CEJ (Table 3).

The safe palatal depth increased from 2 mm to 8 mm from CEJ from first premolar to second molar. The mean safe buccal depth between first and second premolar was 1.83 ± 1.6, 2.42 ± 0.72, 2.9 ± 0.81, and 3.61 ± 0.70 at 2, 4, 6, and 8 mm from CEJ. The safe palatal depth between second premolar and first molar was 1.88 ± 0.88, 2.37 ± 0.80, 2.76 ± 0.83, and 3.35 ± 0.87 at 2, 4, 6, and 8 mm from CEJ. The mean safe palatal depth between first molar and second molar was 2.01 ± 1.05, 2.82 ± 0.89, 3.00 ± 0.77, and 3.56 ± 0.72 at 2, 4, 6, and 8 mm from CEJ. The highest palatal depth was detected between first and second molars at 8 mm depth from CEJ (Table 3).

## 4. Discussion

The aim of this study was to assess the optimal sites of miniscrew insertion in the buccal and palatal alveolar cortical bone in the posterior maxilla by determining cortical bone thickness, interradicular distance, and safe depth of miniscrew insertion using CBCT imaging.

Temporary anchorage devices such as miniscrews are absolute anchors placed in the jaw bone enabling dental movement in orthodontic treatments. In the study of Zheng et al., it was indicated that the success rate of miniscrews could reach up to 80% [1], especially when movement of molar teeth is needed; however, it is strongly recommended to assess the quality and quantity of bone prior to screw insertion. Previous studies have shown 20% injury rate during miniscrew insertion due to inaccuracy of insertion and lack of surgical guidance [18, 19].

In the study of Giudice et al., the most common complication of miniscrew insertion in interradicular spaces was injury to adjacent tooth root, which may further cause pain and inflammation in patients [20].

Watanabe et al. indicated that maxillary miniscrews are significantly more stable than mandibular screws [21]; in addition, they indicated that the distance between screw and root was significantly lower in failure groups. The results of this study indicated that in the buccal region, increase and a subsequent decrease in interradicular space from 2 to 8 mm distance to CEJ in posterior maxilla were detected. The most distant interradicular space was detected between second premolar and first molar at 6 mm distance to CEJ. This result is in consensus with the study of Yoon et al. [22]. Al Amiri et al. also evaluated optimal position of orthodontic miniscrews in the maxilla. Their results indicated that buccally, the interdental bone depth was significantly greater between second premolar and first molar [23]. However, in the study of Liu et al., the best segment for miniscrew insertion was assumed between first and second molars [24]. In this study, the least distance between palatal roots was detected at 2 mm from CEJ between first molar and second molars; however, at 4, 6, and 8 mm from CEJ, the distance between first premolar and second premolar teeth was the lowest. The quantity and quality of the maxillary alveolar process is an important factor to decide where to insert the orthodontic mini screws, necessitating careful preoperative evaluation. According to the present study, the buccal bone thickness is not an optimal site for miniscrew placement. The maximum buccal and palatal interradicular distances were between first and second premolars at a depth of 6 mm from CEJ and between second premolar and first molar at a depth of 8 mm from CEJ. Also, comparing bone thickness between buccal interradicular distance, buccal bone thickness, buccal safety depth, palatal interradicular distance, and palatal safety depth of each posterior maxillary tooth with adjacent tooth at different depths of 2, 4, 6, and 8 mm from CEJ and comparison in depths of 2, 4, 6, and 8 mm of CEJ between each interdental area, a significant difference (*p* value <0.05) between 264 areas was detected. However, in this comparison, between 188 regions, no significant difference (*p* value >0.05) was observed. In another study, miniscrew implantation guided by stereolithographic surgical stent based on CBCT-derived 3D images was performed. The results suggested no root damage in the stent group. However, without a surgical stent, four cases out of 10 miniscrews contacted tooth roots. This study concluded that when facing limited interradicular distance, multiple impacted teeth, and extended maxillary sinus, CBCT-guided techniques are strongly suggested [18]. Marquezan et al. indicated that cortical bone thickness can influence primary miniscrew stability [25]. In this study, cortical bone thickness was the highest on second premolar at 2–6 mm from CEJ and further on second molar from 6 to 8 mm from CEJ. Thinner cortical bone may negatively influence the primary screw stability. However, the intrabony depth is another influencing factor affecting primary stability of miniscrews. In this study, the highest buccal and palatal depth is detected between first molar and second molar. Jin et al. indicated that regardless of the cortical bone density, more energy was required to remove the miniscrews as the implantation depth increased, indicating higher resistance and less risk of falling out [10].

## 5. Conclusion

The highest distance between buccal and palatal roots of posterior maxilla was detected between first and second premolars at 6 mm distance from CEJ and between second premolar and first molar at 8 mm distance from CEJ, respectively. Therefore, it can be assumed that the most optimal sites for miniscrew insertion in the maxilla is nearly at the midroot site of the premolar-molar area.

### 5.1. Limitations and Suggestions

It is important to know that the thickness of the gingival soft tissue may also affect the prognosis of the miniscrews; therefore, further studies are recommended to take this factor into account. Future studies may also evaluate larger sample sizes and assess the relationship between systemic diseases, drug consumption, aging, and craniofacial anomalies on quantitative assessments of both maxilla and mandible arches.

## Figures and Tables

**Table 1:** Distance between buccal and palatal roots in posterior maxilla in millimeter.

Distance between buccal roots								
Distance from CEJ	2 mm		4 mm		6 mm		8 mm	
Teeth	Mean	SD	Mean	SD	Mean	SD	Mean	SD

Canine-1^st^ P	2.52	0.86	2.72	0.81	2.87	0.90	2.73	1.38
1^st^ P-2^nd^ P	2.75	0.83	3.01	0.80	3.28	0.94	2.77	1.50
2^nd^ P-1^st^ M	2.71	0.80	2.79	0.84	2.87	0.94	2.78	1.15
1^st^ M-2^nd^ M	2.36	0.91	2.44	0.85	2.02	1.09	1.64	1.11

Distance between palatal roots								
Distance from CEJ	2 mm		4 mm		6 mm		8 mm	
Teeth	Mean	SD	Mean	SD	Mean	SD	Mean	SD

1^st^ P-2^nd^ P	3.10	0.59	3.46	0.69	3.48	0.89	3.64	0.81
2^nd^ P-1^st^ M	3.68	0.90	4.40	1.09	5.01	1.08	5.30	1.03
1^st^ M-2^nd^ M	2.92	0.99	3.52	0.94	3.88	1.17	4.15	0.95

CEJ, cementoenamel junction; M, molar; mm, millimeter; P, premolar; SD, standard deviation.

**Table 2:** Buccal cortical bone thickness in millimeter.

Buccal cortical bone thickness								
Distance from CEJ	2 mm		4 mm		6 mm		8 mm	
Teeth	Mean	SD	Mean	SD	Mean	SD	Mean	SD
Canine	0.76	0.31	1.04	0.44	0.80	0.54	0.70	0.50
1^st^ P	0.58	0.53	0.86	0.52	0.61	0.41	0.52	0.37
2^nd^ P	1.05	0.96	1.56	0.81	1.23	0.71	0.95	0.77
1^st^ M	0.46	0.54	0.71	0.46	0.53	0.53	0.47	0.49
2^nd^ M	0.78	0.42	1.54	0.70	1.33	0.80	1.19	0.96

CEJ, cementoenamel junction; M, molar; mm, millimeter; P, premolar; SD, standard deviation.

**Table 3:** Safe buccal and palatal bone thickness in millimeter.

Safe buccal depth								
Distance from CEJ	2 mm		4 mm		6 mm		8 mm	
Teeth	Mean	SD	Mean	SD	Mean	SD	Mean	SD

Canine-1^st^ P	1.62	0.69	1.96	0.70	1.72	1.80	1.56	1.50
1^st^ P-2^nd^ P	2.24	1.10	2.52	0.84	2.11	2.30	1.81	1.90
2^nd^ P-1^st^ M	2.37	0.93	2.59	0.81	2.16	2.15	2.06	2.00
1^st^ M-2^nd^ M	2.56	0.80	2.61	1.03	2.50	2.50	2.45	2.50

Safe palatal depth								
Distance from CEJ	2 mm		4 mm		6 mm		8 mm	
Teeth	Mean	SD	Mean	SD	Mean	SD	Mean	SD

1^st^ P-2^nd^ P	1.83	1.06	2.42	0.72	2.90	0.81	3.61	0.70
2^nd^ P-1^st^ M	1.88	0.88	2.37	0.80	2.76	0.83	3.35	0.87
1^st^ M-2^nd^ M	2.01	1.05	2.82	0.89	3.00	0.77	3.56	0.72

CEJ, cementoenamel junction; M, molar; mm, millimeter; P, premolar; SD, standard deviation.

## Data Availability

The datasets used and/or analyzed during the current study are available from the corresponding author upon request.
